# The relationship between amyloid burden, hippocampus, and cholinergic integrity

**DOI:** 10.1002/alz.084746

**Published:** 2025-01-09

**Authors:** Li‐Hua Lee

**Affiliations:** ^1^ Cardinal Tien Hospital, Taipei, Taipei Taiwan

## Abstract

**Background:**

Hippocampal atrophy and degeneration of cholinergic pathways are prominent neuroimaging findings in early Alzheimer’s disease (AD). However, whether these image findings are related to amyloid accumulation is still debated.

**Method:**

From 2021 to 2023, we recruited 116 participants from memory clinic in Cardinal Tien Hospital, Taipei, Taiwan. All our participants received complete neuropsychological tests, 3.0 T brain MRI and amyloid positron emission tomography (PET). Hippocampal volumes were measured through high‐resolution T1 weighted images. DTI metrics such as MD (mean diffusivity) or FA (fractional anisotropy) values were derived from DSI studio. In this study, we use cingulum bundle, external capsule and uncinate fasciculus to simulate cholinergic pathway.

**Result:**

Our study included 41 healthy individuals, 61 with mild cognitive impairment (MCI), and 14 with mild dementia. Significant differences were observed across these groups in hippocampal volumes, MD values of cholinergic tract, and amyloid SUVR values. Our linear regression analysis, which adjusted for variables including sex, age, and education level, demonstrated that these three neuroimaging markers are significant predictors of cognitions. Notably, the analysis revealed that hippocampal volume, amyloid SUVR and the MD values of the cholinergic pathways independently predict the severity of cognition. When evaluating the association between amyloid SUVR, hippocampal volume and cholinergic integrity by multilinear regression models which were controlled with age, sex, and presence of APOE4 carrier status, we found that hippocampal volume is significantly associated with the amyloid burden (β = −0.215; p = 0.022), while cholinergic MD values are not related to amyloid SUVR.

**Conclusion:**

Amyloid accumulation is associated with hippocampal atrophy, but not cholinergic integrity in our study. The impairment of cholinergic integrity may be due to etiologies other than amyloid. Neuroimaging markers focusing on cholinergic integrity should be closely observed independently from others. Multitargeting treatments, in addition to anti‐amyloid, may be required for better efficacy in the prevention and treatment of cognitive decline in AD.

